# Modeling of Endothelial Calcium Responses within a Microfluidic Generator of Spatio-Temporal ATP and Shear Stress Signals

**DOI:** 10.3390/mi12020161

**Published:** 2021-02-07

**Authors:** Yong-Jiang Li, Miao Yu, Chun-Dong Xue, Hai-Jun Zhang, Guo-Zhen Wang, Xiao-Ming Chen, Kai-Rong Qin

**Affiliations:** 1 School of Optoelectronic Engineering and Instrumentation Science, Dalian University of Technology, Dalian 116024, China; yongjiangli@dlut.edu.cn (Y.-J.L.); xuechundong@dlut.edu.cn (C.-D.X.); guozhen_0510@163.com (G.-Z.W.); 2 School of Biomedical Engineering, Dalian University of Technology, Dalian 116024, China; yumiao@mail.dlut.edu.cn (M.Y.); haijunzhang@mail.dlut.edu.cn (H.-J.Z.)

**Keywords:** intracellular calcium dynamics, spatio-temporal signals, ATP and shear stress stimuli, theoretical modeling, endothelial cells

## Abstract

Intracellular calcium dynamics play essential roles in the proper functioning of cellular activities. It is a well known important chemosensing and mechanosensing process regulated by the spatio-temporal microenvironment. Nevertheless, how spatio-temporal biochemical and biomechanical stimuli affect calcium dynamics is not fully understood and the underlying regulation mechanism remains missing. Herein, based on a developed microfluidic generator of biochemical and biomechanical signals, we theoretically analyzed the generation of spatio-temporal ATP and shear stress signals within the microfluidic platform and investigated the effect of spatial combination of ATP and shear stress stimuli on the intracellular calcium dynamics. The simulation results demonstrate the capacity and flexibility of the microfluidic system in generating spatio-temporal ATP and shear stress. Along the transverse direction of the microchannel, dynamic ATP signals of distinct amplitudes coupled with identical shear stress are created, which induce the spatio-temporal diversity in calcium responses. Interestingly, to the multiple combinations of stimuli, the intracellular calcium dynamics reveal two main modes: unimodal and oscillatory modes, showing significant dependence on the features of the spatio-temporal ATP and shear stress stimuli. The present study provides essential information for controlling calcium dynamics by regulating spatio-temporal biochemical and biomechanical stimuli, which shows the potential in directing cellular activities and understanding the occurrence and development of disease.

## 1. Introduction

Calcium ion (Ca^2+^) is a highly universal second messenger that controls a wide variety of cellular functions starting from cell birth to apoptosis [1,2,3,4,5,6]. Ca^2+^ mediates these functions by distinct Ca^2+^ signal transduction pathways that translate Ca^2+^ signals with varying spatio-temporal behaviors into specific biological activities, such as insulin secretion, muscle movement, heart beats, or brain information processing [7,8,9]. In physiological states, it is well known that Ca^2+^ signals are regulated by the spatio-temporal physiochemical extracellular microenvironment, wherein physiochemical cues are spatially varying in magnitude and frequency. Upon stimulation of these physiochemical signals, the modulating signals are encoded in the complex spatio-temporal dynamics of cytosolic Ca^2+^ concentration, ranging from calcium spiking and oscillations to complex Ca^2+^ waves, which ultimately lead to the diversity and specificity of cellular behaviors as well as dysfunctions [10,11,12,13,14,15]. Specifically, angiogenesis, the formation of new blood vessels, is highly correlated to the intracellular Ca^2+^ dynamics in vascular endothelial cells (VECs) [14,15,16,17,18,19,20,21], which is coordinated by the spatial connectivity between soluble biochemical agonists (e.g., growth factors, ATP) and biomechanical forces (e.g., shear stress) generated by pulsatile blood flow. Exposed to spatial-temporal growth factors, VECs exhibit spatial variation in Ca^2+^ microdomains and Ca^2+^ waves. Unimodal and oscillatory Ca^2+^ responses can respectively contribute to cell migration and cell proliferation [15,19,20,21], which are involved at critical phases of angiogenesis. Therefore, it is inferred that the control of Ca^2+^ responses by regulating spatio-temporal physiochemical stimuli is crucial for ensuring the proper functioning of cellular activities and biological processes.

In the past decades, extensive works on Ca^2+^ signal transduction pathways and corresponding regulation mechanisms have been carried out. Due to the complexity of human body and the existence of various interference factors, the quantitative characterization of Ca^2+^ responses in vivo is challenging. Therefore, a lot of efforts were made in vitro to mimic the spatio-temporal physiochemical extracellular microenvironment, and to probe Ca^2+^ responses induced by the spatial-temporal physiochemical stimuli. Recently, with the developments in microfabrication and microfluidic techniques, the microfluidic system has become an ideal platform for quantitatively probing Ca^2+^ dynamics in response to biochemical and biomechanical stimuli [11,22,23,24,25]. Substantial studies have been conducted to control Ca^2+^ dynamics through the stimulation of extracellular biochemical (e.g., growth factors [15,18], ATP [24,25,26] and glucose [7]) or biomechanical (e.g., shear stress [27], electromagnetic [28] and optical forces [9]) signals. An example of this is the study carried out by Chen et al. [24,25] in which the temporal Ca^2+^ dynamics induced by various ATP stimulation waveforms were investigated through a microfluidic chemical function generator. It is concluded that temporal features of Ca^2+^ dynamics can be altered by a time-varying ATP waveform. Yamamoto et al. [29] and Jafarnejad et al. [30] demonstrated that flow-induced Ca^2+^ responses indicate a shear stress-dependent manner. Stepwise increasing shear stress results in increasing ATP spikes, which further mediate Ca^2+^ responses in the similar manner. Some researchers also revealed the different regimes of Ca^2+^ signaling corresponding to the substrate rigidity and ligand concentration at different levels [26,31]. However, most studies have only focused on biochemical stimuli or biomechanical stimuli. The flexible and precise control of biochemical and biomechanical stimuli are still challenging. Even some microfluidic systems can replicate and control physiochemical signals in complex waveforms, the transmission of signals within microchannels is rarely considered, which greatly influence the spatial distribution of physiochemical signals [32,33].

Along with experimental approaches, theoretical modelings have been a valuable method for understanding Ca^2+^ signaling pathways and making substantial predictions. Considering diverse Ca^2+^ signaling pathways and extracellular physiochemical stimuli, the multiple Ca^2+^ signal patterns observed at the single cell level have been finely modeled, including calcium spikes, regular oscillatory, and complex waves [31,34,35,36,37,38,39,40,41,42,43]. The Ca^2+^ responses to continuous and pulsed biochemical stimulations in both the oscillatory (low ligand concentration) and acute (high ligand concentration) regimes have been analyzed. The Ca^2+^ responses synchronized to periodic stimuli (phase-locking feature) have also been theoretically proved, as well as the dependence of Ca^2+^ oscillatory frequency on stimulation concentrations [31,34,35]. Qin et al. [36,37,38] have reported a Ca^2+^ signaling model elicited by the activations of both ATP and shear stresses. The results reveal the fidelity of Ca^2+^ responses to stepwise increasing shear stress combined with ATP stimuli. The frequency of Ca^2+^ oscillatory increases with the increase in ATP concentration. These theoretical studies provide new insights into Ca^2+^ signaling pathways and the corresponding regulation factors. Nevertheless, based on our knowledge, the existing models at the single cell level focus on Ca^2+^ dynamics regulated by temporal physiochemical signals, the spatial characteristics of extracellular stimuli are often ignored. In addition, little information exists on how spatio-temporal and amplitude varying extracellular stimuli modulate Ca^2+^ response, and how Ca^2+^ signals encode the frequency and amplitude information of modulating signals are not clear.

In the present study, we construct a theoretical model to investigate the effects of spatio-temporal coupling biochemical and biomechanical signals on the intracellular Ca^2+^ responses in VECs. ATP and shear stress, the important and ubiquitous extracellular stimuli of VECs [24,25,26,27,37,44], are respectively selected as the biochemical and biomechanical stimuli. Based on a microfludic generator of spatio-temporal ATP and shear stress signals, a theoretical model is established which combines extracellular mass transport and intracellular Ca^2+^ dynamics induced by both ATP and shear stress stimuli. By considering the transmission characteristics of microchannels, the capacity of the microfluidic generator and the transmission of ATP signal within the microchannel are analyzed. Exposed to the transmitted spatio-temporal ATP and shear stress stimuli, the intracellular Ca^2+^ dynamics of adherently cultured cells are theoretically investigated. The influence of spatio-temporal characteristics of ATP and shear stress stimuli on the intracellular Ca^2+^ responses are discussed. Consequently, this study provides crucial information needed to regulate intracellular Ca^2+^ dynamics for further controlling cellular functions and biological activities.

## 2. Mathematical Methods

### 2.1. Microfluidic Chip and Model Development

**Figure 1:**
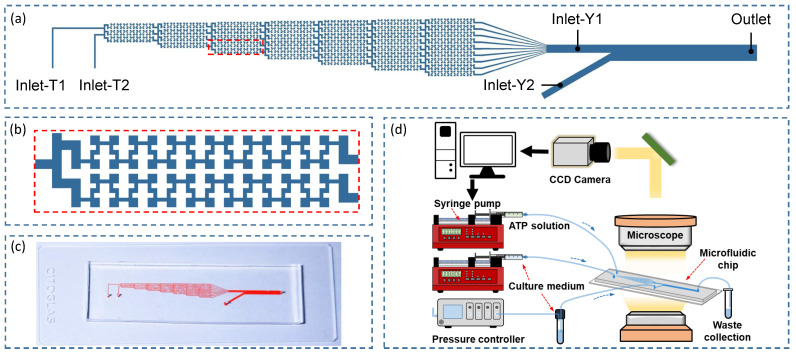
Microfluidic chip for generating spatio-temporal ATP and shear stress signals (**a**–**c**) and microfluidic system (**d**) for probing intracellular Ca^2+^ dynamics [46].

In this study, the theoretical modeling is established based on a developed microfluidic generator of biochemical and biomechanical signals for probing cellular activities [45,46] (Figure 1). The microfluidic system (Figure 1d) consists of a microfluidic chip, peripheral flow pumps (Ne-1000, ERA, Farmingdale, NY, USA) and a microscope (IX73, Olympus, Tokyo, Japan) with a CCD camera (DS126431, Canon, Tokyo, Japan). Among these, the microfluidic chip is the key component for signals generation and cell culture (Figure 1a). It integrates a “Christmas tree” structure as one inlet (Inlet-Y1) of a Y-shaped microchannel, into which an ATP solution and ATP free cell culture medium (referred to as perfusion solution) are introduced at the same flow rate, a linear concentration profile at Inlet-Y1 is initially created through free diffusion and convection [45,47]. The other inlet (Inlet-Y2) of the Y-shaped microchannel is connected with a pressure-driven pump (OB1 MK3, Elveflow, Paris, France) for generating dynamic flow in arbitrary waveform. Due to the low Reynolds number and laminar characteristic of flow, the time-varying flow introduced into Inlet-Y2 can regulate and produce spatio-temporal ATP and shear stress signals within the main channel of the Y-shaped microchannel, wherein scattered VECs are cultured at the bottom of the microchannel. Thus, this microfluidic setup provides an ideal platform for probing cellular activities (e.g., intracellular Ca^2+^ dynamics) in response to spatio-temporal ATP and shear stress signals. It should be noted that the theoretical modeling is nevertheless developed based on this proposed microfluidic system, the modeling situation can be generally applicable to universal microfluidic systems [24,25,48,49].

### 2.2. Mathematical Modeling

#### 2.2.1. Sub-Model of Extracellular ATP Transport

**Figure 2:**
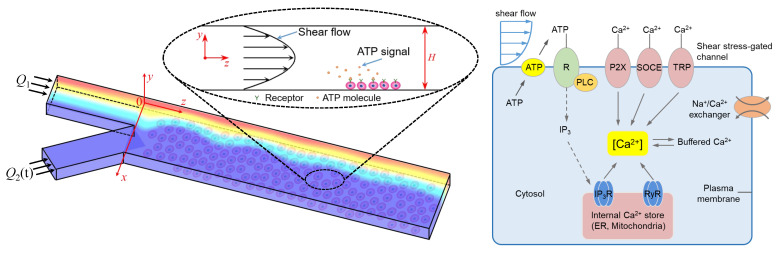
Schematic of VECs exposed to ATP and shear stress stimuli within the microchannel (**left**) and Ca^2+^ signaling pathways activated by ATP and shear stress signals (**right**).

Based on the proposed microfluidic system, an initial linear ATP concentration profile is introduced into Inlet-Y1 at a constant flow rate $Q_{1}$, and Inlet-Y2 is introduced with perfusion solution at a pulsatile flow rate $Q_{2}\left(t\right)$ (Figure 2). Within the main microchannel, the two side-by-side steams of flow are the Newtonian fluids that have fully developed to laminar flows. Under these assumption, neglecting the inlet effect, the stream widths of the two solutions are proportional to the corresponding flow rates [32].

(1) $$\frac{W_{1}\left(t\right)}{W_{2}\left(t\right)}=\frac{Q_{1}}{Q_{2}\left(t\right)}$$

where $W_{1}\left(t\right)$ and $W_{2}\left(t\right)$ accordingly denote the steam widths of ATP and perfusion solution. In this study, the flow rate ratio is defined as $Q_{1}/Q_{2}\left(t\right)=\varepsilon \left(t\right)/\left(1-\varepsilon \left(t\right)\right)$. Hence, the width of ATP solution is ε(t)W, which initially determines the ATP distribution in the main microchannel.

Simultaneously, the soluble ATP molecules are convected and diffused with the flow, wherein the concentration of diffusible ATP ϕ(x,y,z,t) is governed by the convection–diffusion equation

(2) $$\frac{\partial \phi }{\partial t}+u\left(y,t\right)\frac{\partial \phi }{\partial z}=D\nabla^{2}\phi$$

where *D* is the diffusivity of ATP molecule and $\nabla^{2}$ represents the three-dimensional Laplacian. With the quasi-steady flow assumption and the consideration of the pulsatile flow characteristics in the shallow microchannel, the velocity profile u=u(y,t) can be given by

(3) $$u\left(y,t\right)=\frac{3}{2}\left[1-{\left(\frac{2y}{H}\right)}^{2}\right]\bar{u}\left(t\right)=\frac{3}{2}\left[1-{\left(\frac{2y}{H}\right)}^{2}\right]\frac{Q(t)}{HW}$$

where $\bar{u}\left(t\right)$ is the average velocity, and $Q\left(t\right)=Q_{1}+Q_{2}\left(t\right)$ is the total flow rate.

Based on the quasi-steady assumption [50], the shear stress exerted to VECs at the bottom of the microchannel (Figure 2) can be given by

(4) $$\tau_{w}=\eta \frac{\partial u(y,t)}{\partial y}{|}_{y=-H/2}=\frac{6\eta \bar{u}\left(t\right)}{H}=\frac{6\eta Q(t)}{WH^{2}}$$

where η is the fluid viscosity.

By considering the time-dependent Taylor–Aris dispersion and taking the average along heightwise, the governing equation of ATP concentration (Equation (2)) can be thus approximated as [32]

(5) $$\frac{\partial \bar{\phi }}{\partial t}+\bar{u}\left(t\right)\frac{\partial \bar{\phi }}{\partial z}=D\frac{\partial^{2}\bar{\phi }}{\partial x^{2}}+D_{eff}\frac{\partial^{2}\bar{\phi }}{\partial z^{2}}$$

$D_{eff}$ is the effective diffusivity coefficient that is expressed by

(6) $$D_{eff}=D+D_{T}=D\left[1+\frac{Pe^{2}}{210}{\left(\frac{H}{W}\right)}^{2}\right]$$

where $Pe=\bar{u}\left(t\right)W/D$ is the Peclet number, which describes the ratio of convection to diffusion of a solute.

According to the experimental conditions, the boundary conditions of Equation (5) are derived as:

(7) $$\begin{array}{cccc} B.C.1: & \bar{\phi }\left(x,z,t\right){|}_{z=0}=\phi_{0}\left(1-\frac{x}{\varepsilon (t)W}\right), & 0\leq x\leq \varepsilon (t)W & \\ B.C.2: & \bar{\phi }\left(x,z,t\right){|}_{z=0}=0, & \varepsilon (t)W<x\leq W & \\ B.C.3: & \partial \bar{\phi }{/\partial z|}_{z\to \infty }=0, & \partial \bar{\phi }{/\partial x|}_{x=0}=0, & \partial \bar{\phi }{/\partial x|}_{x=W}=0 \end{array}$$

where $\phi_{0}$ is the constant concentration of ATP solution introduced into Inlet-T1.

#### 2.2.2. Sub-Model of Intracellular Calcium Dynamics

Induced by the spatio-temporal extracellular ATP and shear stress (see Figure 2), the intracellular Ca^2+^ dynamics of adherently cultured VECs is theoretically modeled. As shown in Figure 2, the model includes not only Ca^2+^ signaling pathways through P2Y receptors and P2X_4_ Ca^2+^ channels (indirect mechanism) [29,38] but also Ca^2+^ signaling induced by shear stress through TRPV_4_ and/or TRPV_4_-C_1_ compound channels in VECs (direct mechanism) [38,40]. Extracellular ATP, shear stress, and ATP induced by shear stress are primary stimulation sources of adherent VECs. The contribution of ATP hydrolysis products to Ca^2+^ change is negligible due to the minimal effect. According to these conditions, intracellular calcium homeostasis is maintained by balancing multiple Ca^2+^ signaling cues, including the Ca^2+^ release from internal calcium stores to the cytosol, the Ca^2+^ restore into calcium stores from the cytosol, the Ca^2+^ inflow from extracellular fluid into the cytosol, the extrusion and exchange of Ca^2+^ to the extracellular environment, and the Ca^2+^ combined with soluble cytosolic proteins. The dynamic of cytosolic-free calcium ion can be thus described as

(8) $$\frac{dC}{dt}=q_{rel}-q_{res}+q_{in}-q_{out}-q_{buf}$$

where *C* is the cytosolic-free Ca^2+^ concentration, $q_{rel}$ represents the release rate of Ca^2+^ from the calcium stores, $q_{res}$ represents the restore rate of Ca^2+^ back into calcium stores from the cytoplasm, $q_{in}$ stands for Ca^2+^ inflow through Ca^2+^ channels, $q_{out}$ stands for Ca^2+^ outflow to the extracellular environment refer to the Ca^2+^ clearance mechanism, and $q_{buf}$ denotes the buffering rate of Ca^2+^ by soluble cytosolic proteins.

Based on the existing models [36,37,38,39,40,51], the expressions of the four fluxes i.e., $q_{rel}$, $q_{res}$, $q_{in}$, $q_{out}$ and $q_{buf}$ in Equation (8) are as follows:

(9) $$q_{rel}=k_{3}\frac{C}{K_{CICR}+C}{\left(\frac{i}{i+K_{2}}\right)}^{3}C_{s},$$

(10) $$q_{res}=k_{4}{\left(\frac{C}{K_{3}+C}\right)}^{2}-k_{5}C_{s}^{2}$$

(11) $$q_{out}=\frac{k_{8}C}{K_{4}+C}$$

(12) $$q_{buf}=k_{6}C\left(B_{T}-C_{b}\right)-k_{7}C_{b}$$

where *i* is the concentration of IP_3_, an intracellular signaling mediator. $C_{s}$ is the Ca^2+^ concentration in the calcium stores, and $C_{b}$ is the concentration of cytosolic buffering Ca^2+^. $K_{CICR}$ represents the sensitivity of calcium stores in the Ca^2+^-induced Ca^2+^ release (CICR) mechanism. $K_{2}$, $K_{3}$, $K_{4}$ are the Michaelis–Menten constants, and $B_{T}$ is the total concentration of Ca^2+^ buffering sites on proteins in the cytosol. $k_{3}$ and $k_{4}$ are accordingly the release and restore rate of calcium stores, $k_{5}$ is the leak rate of Ca^2+^, $k_{6}$ and $k_{7}$ are the buffering and debuffering rate constants, respectively, and $k_{8}$ is the maximum Ca^2+^ efflux of the Ca^2+^ clearance mechanism.

Similarly, the balance of Ca^2+^ in calcium stores and buffering proteins are governed by

(13) $$\frac{dC_{s}}{dt}=\frac{V_{c}}{V_{s}}\left(q_{res}-q_{rel}\right)$$

(14) $$\frac{dC_{buf}}{dt}=q_{buf}$$

where $\frac{V_{c}}{V_{s}}$ is the volume ratio of cytosol to calcium stores. The initial conditions of $C_{s}$ and $C_{buf}$ are as follows:

(15) $$C_{b}\left(0\right)=C_{b0}=k_{6}C_{0}B_{T}\left(k_{7}+k_{6}C_{0}\right)$$

(16) $$C_{s}\left(0\right)=C_{s0}=\frac{{\left(\frac{k_{4}}{k_{5}}\right)}^{2}C_{0}}{K_{3}+C_{0}}$$

As shear flow with soluble ATP molecules are transported to cells cultured at the bottom of the microchannel (see Figure 2), cells can sense the biochemical (i.e., ATP) and biomechanical stimuli (i.e., shear stress) and transduce them into downstream Ca^2+^ signaling pathways, such as the synthesis of IP_3_ and the Ca^2+^ influx through Ca^2+^ channels. Among these processes, it is well known that the concentration of IP_3_ as a second messenger is dependent on the extracellular ATP concentration, the relationship can be expressed by [36,51,52]

(17) $$\frac{di}{dt}=k_{1}\frac{\phi }{K_{c}+\phi }{|}_{y=0}\frac{C}{K_{1}+C}-k_{2}i$$

where $k_{1}$ and $k_{2}$ are the production and degradation rates of IP_3_, respectively. $K_{1}$ and $K_{c}$ are the Michaelis–Menten constants.

In addition, the extracellular ATP can directly activate Ca^2+^ influx through P2X_4_ channels [36], and the influx rate of Ca^2+^ influx through P2X_4_ channels $q_{p2x4}$ is presented as

(18) $$q_{in\_p2x4}=k_{p2x4}{\left(\frac{\phi }{K_{p2x4}+\phi }\right)}^{3}\left(C_{ex}-C\right)$$

where $k_{P2X4}$ is the calcium ion flux rate, $K_{\phi }$ is the Michaelis–Menten constant for the interaction between ATP and P2X_4_, and $C_{ex}$ is the concentration of extracellular calcium ion.

Except for ATP-gated P2X_4_ channels, calcium ion transmembrane transport, denoted by $q_{in}$ in Equation (8), is mainly through the TRP channels (mechanosensitive TRPV_4_ and TRPC_1_ compound channels). Therefore, $q_{in}$ is given in the following form:

(19) $$q_{in}=q_{in\_p2x4}+q_{in\_TRP}+q_{in\_pass}$$

where $q_{in\_pass}$ is a constant reflecting the passive influx of Ca^2+^ in the no-load case. $q_{in\_TRP}$ represents the Ca^2+^ inflow rate through the TRP channels. The TRPVC_4_ and TRPC_1_ channels are both mechanosensitive, which can be directly activated by shear stress. For ease of reference, the expressions of Ca^2+^ inflow through TRPV_4_ and TRPC_1_ channels are given by the following form [38]:

(20) $$q_{in\_TRP}=q_{max}p_{1}p_{2}p_{3}\left(C_{s0}-C_{s}\right)\left(C_{ex}-C\right)$$

where $q_{max}$ is the rate constant, which represents the maximum Ca^2+^ inflow when all channels are open. The parameter $p_{1}$ describes the direct effect of shear stress and probability of the open state of the TRPV_4_ channels induced by shear stress, which is expressed as [40]

(21) $$p_{1}=\frac{1}{1+\alpha \cdot \exp\left(\frac{-f_{e}W\left(\tau_{w}\right)}{8kTN}\right)}$$

where α is a positive constant, ${\left(1+\alpha \right)}^{-1}$ is the open probability of a channel in the no-load case. $f_{e}\left(f_{e}\in \left[0,1\right]\right)$ is the fraction of the energy within the membrane that gates the shear stress-sensitive Ca^2+^ channels, *k* is the Boltzmann constant, *T* is the temperature, and *N* is the Ca^2+^ channels density per unit area. $W_{\tau_{w}}$ is the strain energy density activated by shear stress, it can be expressed as

(22) $$W_{\tau_{w}}=\frac{{\left(\varepsilon \tau_{w}l+\sqrt{16\delta^{2}+\varepsilon^{2}\tau_{w}^{2}l^{2}}-4\sigma \right)}^{2}}{\left(\varepsilon \tau_{w}l+\sqrt{16\delta^{2}+\varepsilon^{2}\tau_{w}^{2}l^{2}}\right)}$$

where $\varepsilon \left(\varepsilon \in \left[0,1\right]\right)$ is the fraction of the applied load borne by the plasma membranes, *l* is the length of the cell in the flow direction, and δ is the shear modulus of the cell membrane.

The parameter $p_{2}$ describes the probability of the open state of the TRPV_4_-C_1_ channels activated by the binding of three IP_3_ molecules to IP_3_R type 3 in the membrane of the Ca^2+^ stores, which is presented as

(23) $$p_{2}=b_{1}\left(1+a_{1}\cdot \frac{i^{3}}{{\left(K_{i}+i\right)}^{3}}\right)$$

where $K_{i}$ is the Michaelis–Menten constant, and $a_{1}$ and $b_{1}$ are positive constants.

The parameter $p_{3}$ stands for the probability of the open state of the TRPV_4_-C_1_ channels decayed by intracellular Ca^2+^ concentration, which satisfies the following exponential function,

(24) $$p_{3}=c_{1}+c_{2}e^{-c_{3}*C}$$

where $c_{1}$, $c_{2}$, and $c_{3}$ are positive constants.

#### 2.2.3. Model Parameters and Simulation Methods

Dimensions of the microfluidic chip and default parameters used in the sub-model of extracellular ATP transport are listed in Table 1. The dimensions’ parameters are the same as those of the microfluidic chip. The parameter values for intracellular Ca^2+^ dynamics model are sampled similarly from the previous models [38,51]. All of the mathematical simulations are coded in MATLAB (MathWorks 2020a). The sub-model of extracellular ATP transport (Equations (5) and (7)) is solved by a finite difference method. The emerging temporal ATP and shear stress at observing points are then taken as inputs of sub-model of intracellular Ca^2+^ dynamics, where the system of ordinary differential equations (ODEs) is solved with the stiff solver ode15s.

## 3. Results

### 3.1. Generation and Transport of ATP Signals

**Figure 3:**
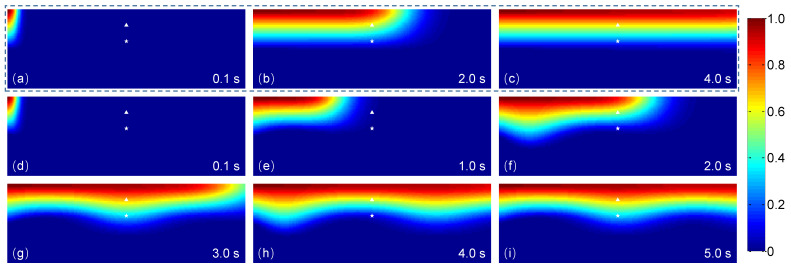
Distributions of ATP concentration within the main channel of the Y-shaped microchannel at different intervals for a steady $Q_{2}=Q_{1}$ (**a**–**c**) and a pulsatile perfusate flow $Q_{2}=Q_{1}\times \left(1+0.5\sin\left(\pi t\right)\right)$ (**d**–**i**) introduced into Inlet-Y2, respectively. Triangle and star points indicate the observation positions at x=1/5W and x=2/5W for z=L.

**Figure 4:**
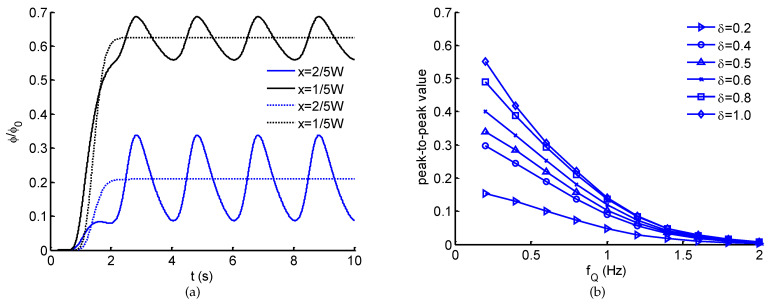
(**a**) Time evolutions of ATP concentrations at the observation points corresponding to the steady (dashed lines) and pulsatile (solid lines) pattern of perfusion solution in Figure 3. (**b**) The peak-to-peak values of emerging ATP signals vary with pulsatile frequency $f_{Q}$ and pulsatile amplitude δ of the perfusion solution.

The generation of spatio-temporal ATP in the microchannel is demonstrated in Figure 3. In the case of steady flows (Figure 3a–c), a solution with linearly spatial ATP concentration and a perfusion solution at the same rate $Q_{1}=Q_{2}$ are accordingly introduced into Inlet-Y1 and Inlet-Y2. With the convection and free diffusion of flows, a steady spatial ATP distribution is generated after ∼4 s (Figure 3c), showing a clearly linear ATP profile along the transverse direction. In the flow direction, a slight change in ATP profile due to the transverse diffusion. When the steady perfusion solution is altered to a pulsatile pattern, based on the characteristics of laminar flow, a spatio-temporal ATP concentration distribution can be created. As indicated in Figure 4a, dynamic ATP signals are generated with similar waveform and frequency of the sinusoidal perfusate flow. Furthermore, at different observation points, the emerging ATP signals show the difference in average and peak-to-peak value. The average of emerging ATP signals is primarily dominated by the transversal position, which is approximate to the ATP concentration when both flows are in steady forms (Figure 4a). However, the peak-to-peak values of emerging ATP signals exhibit the dependence on location (Figure 4a), pulsatile frequency $f_{Q}$ as well as pulsatile amplitude δ (Figure 4b). For a fixed pulsatile amplitude, the peak-to-peak values of emerging ATP signals are attenuated with the increasing frequency, demonstrating an equivalent low-pass filtering effect of microchannels [32,33,38]. For this reason, it can be concluded that a perfusion solution pulsating at high frequency is detrimental to the generation and transportation of ATP signals. To make up the inherent disadvantage, an alternative approach would be to enlarge the pulsatile amplitude (Figure 4b). It should be noted that pulsatile flow greatly affects the nonlinear modulation on the transport of biochemical signals. The increase of pulsatile amplitude will enhance the nonlinear modulation effect [33]. To precisely control spatio-temporal ATP in the microchannel, multiple factors should be considered and balanced.

Except for the spatio-temporal ATP signals, within the microchannel, a pulsatile shear stress originated from the pulsatile flow is uniformly distributed simultaneously. The shear stress is proportional to the total flow rate Q(t) indicated by Equation (4), thus it varies at the same frequency of the pulsatile flow $f_{Q}$. Therefore, cells cultured at the bottom of the microchannel are exposed to spatio-temporal ATP and shear stress signals. On the side of the ATP solution, cells are stimulated by shear stress coupled with temporal ATP signals of different amplitudes, while, on the side of perfusion solution, cells are exposed to shear stress alone. In brief, the proposed microfluidic chip is capable for generating spatio-temporal ATP and shear stress signals, which facilitates the study of cellular functions induced simultaneously by multiple combinations of biochemical and biomechanical stimuli.

### 3.2. Model Validation of Calcium Dynamics

**Figure 5:**
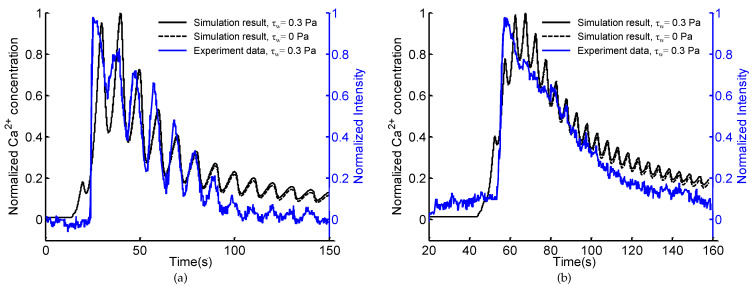
Comparisons of simulation results and experimental results [25] of intracellular Ca^2+^ concentrations induced by sinusoidal ATP signals of 0.1 Hz (**a**) and 0.2 Hz (**b**) coupled with constant shear stress $\tau_{w}=0.3$ Pa.

Prior to investigating Ca^2+^ dynamics induced by the spatio-temporal ATP and shear stress signals, the proposed model of Ca^2+^ dynamics is firstly validated by comparing with the existing experimental results [25]. Under the experimental conditions, cells adherently cultured within the microchannels are exposed to sinusoidal ATP signals of 0.1 Hz and 0.2 Hz coupled with almost invariant shear stress (around 0.3 Pa), the theoretically modeling results are accordingly compared with the experimental results (Figure 5). Exposed to sinusoidal ATP stimulation of 10 μM at 0.1 Hz, the intracellular Ca^2+^ initially increases sharply (around 25 s) and then oscillates for a few minutes with a decreasing plateau level and oscillatory amplitude. The Ca^2+^ oscillation is subject to the extracellular sinusoidal ATP stimulus and varies at the same frequency (∼0.1 Hz) as the ATP waveform. Comparing the modeling and experimental results, the differences in plateau levels and oscillatory amplitudes (after 100 s) may be caused by the nonlinear relation between Ca^2+^ and fluorescence intensity [25]. Except for these differences, the Ca^2+^ concentration predicted by the model (Section 2.2.2) shows great similarity with the experimental data. The low shear stress has no significant influence on Ca^2+^ concentration (Figure 5). As the frequency of the ATP signal increases to 0.2 Hz, the theoretically predicted results are consistent with the experimental one except for the sharp peaks which might be difficult to identify for the detection technique. Briefly, the theoretical model of Ca^2+^ dynamics is capable of simulating cytosolic Ca^2+^ in response to dynamic ATP and shear stress stimuli, which can be an alternative to experimental trials for understanding Ca^2+^ dynamics.

### 3.3. Ca^2+^ Dynamics Regulated by Spatio-Temporal ATP and Shear Stress Stimuli

**Figure 6:**
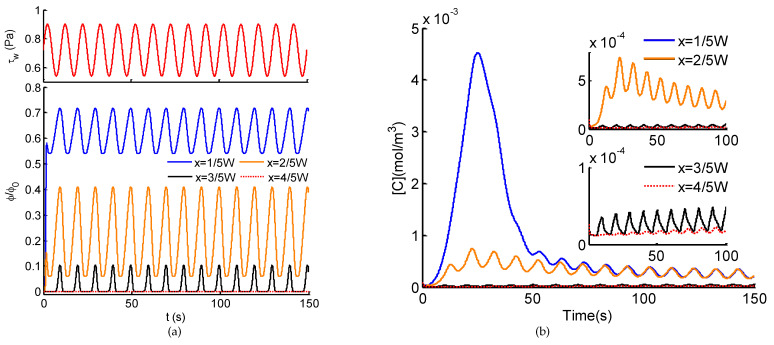
(**a**) Normalized ATP signals in the transverse direction (*x*-axis) and the uniform sinusoidal shear stress $\tau_{w}$ within the microchannel. (**b**) intracellular Ca^2+^ dynamics corresponding to the stimuli of ATP signals couple with sinusoidal shear stress ($f_{Q}=0.1$ Hz) indicated in (**a**).

Taking advantages of the microfluidic system and the Ca^2+^ dynamics model for dynamic stimuli, the Ca^2+^ dynamics induced by the spatio-temporal ATP and shear stress signals are investigated. Figure 6a shows a typical example of emerging spatio-temporal ATP and shear stress signals when the perfusion solution flows at $Q_{2}\left(t\right)$ = 1.5 $\times 10^{-10}\left(1+\delta \cdot \sin(2\pi f_{Q}t)\right)$ m^3^/s, where δ= 0.5, $f_{Q}=$ 0.1 Hz. With the convection and diffusion of flow, on the side of the ATP solution (see lines at x=1/5W and x=2/5W), sinusoidal ATP signals with distinct averages and amplitudes are generated, indicating the same frequency as the sinusoidal flow of $Q_{2}\left(t\right)$. On the contrary, on the side of perfusion solution (see lines at x=3/5W and x=4/5W), a rectified sinusoidal ATP signal appears near the centerline (x=1/2W) while no ATP stimulus arises far away from the ATP solution (x=4/5W). Simultaneously, uniform shear stress in the sinusoidal form of 0.1 Hz (Figure 6a) exerts to cells at the bottom of the microchannel. In brief, at different positions within the microchannel, different combinations of dynamic ATP and shear stress emerge, which further activate distinct cellular Ca^2+^ signaling.

Corresponding to the spatio-temporal ATP and shear stress signals (in Figure 6a), their activations on intracellular Ca^2+^ dynamics of adherently cultured cells are demonstrated in Figure 6b. At different locations, due to the different combinations of ATP and shear stress signals, the resulting Ca^2+^ responses indicate distinct patterns. Induced by the sinusoidal ATP stimulus of high average and low pulsatile amplitude (blue lines in Figure 6), cytosolic Ca^2+^ shows a unimodal mode with weak oscillations in the basal level. Under the same shear stress stimulus, a similar trend is observed for a step ATP signal of ∼6.2 μM (the average of the sinusoidal ATP stimulus). Therefore, at a high ATP level, a weak pulsation of ATP has no influence on cytosolic Ca^2+^, the oscillations is dominated by pulsatile shear stress. With moderate ATP stimulus of high pulsatile amplitude, both pulsatile ATP and shear stress contribute to Ca^2+^ oscillations, leading to synchronous changes in Ca^2+^ responses at the pulsatile frequency of ATP and shear stress. When the ATP stimulus reaches a low level or no ATP stimulus, Ca^2+^ oscillations emerge at the minimal level (subfigure in Figure 6b). However, the addition of ATP stimulus enhances the amplitudes of oscillations compared with the single excitation of shear stress. To summarize, intracellular Ca^2+^ dynamics reveals a significant dependence on the levels of average ATP and pulsatile amplitude. Ca^2+^ oscillations can be activated by both pulsatile ATP and shear stress within a range of stimuli levels.

**Figure 7:**
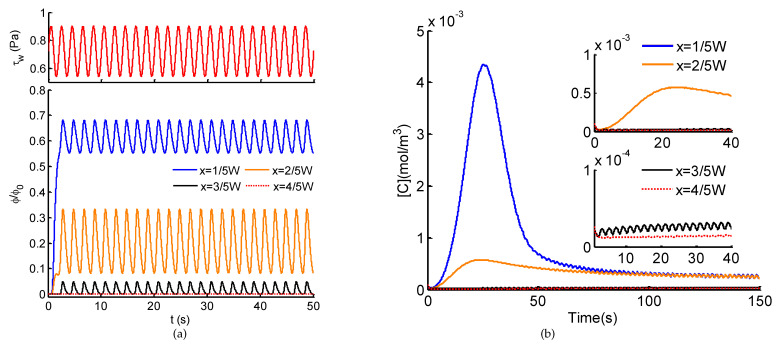
(**a**) Normalized ATP signals in the transverse direction (*x*-axis) and the uniform sinusoidal shear stress $\tau_{w}$ within the microchannel; (**b**) intracellular Ca^2+^ dynamics in response to the stimuli of ATP signals couple with sinusoidal shear stress ($f_{Q}=0.5$ Hz) indicated in (**a**).

**Figure 8:**
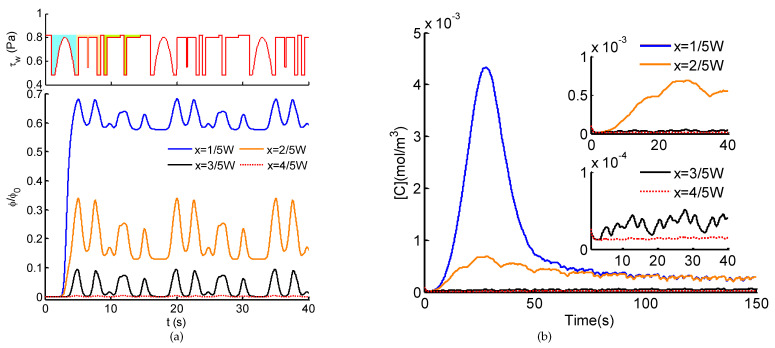
(**a**) Normalized ATP signals in the transverse direction (*x*-axis) and the uniform sinusoidal shear stress $\tau_{w}$ within the microchannel; (**b**) intracellular Ca^2+^ dynamics in response to the stimuli of ATP signals couple with CELL-type shear stress indicated in (**a**).

We further probe the dependence of Ca^2+^ dynamics on ATP and shear stress stimuli at higher frequency ($f_{Q}=0.5$ Hz). Intracellular Ca^2+^ dynamics of adherently cultured cells demonstrate similar tendency to spatio-temporal ATP and shear stress stimuli (Figure 7). The big difference between them (Figure 6 and Figure 7) lies in the Ca^2+^ in response to moderate pulsatile ATP stimulus, the Ca^2+^ oscillations are inhibited at higher pulsatile frequency. In the case of shear stress stimulus alone, the weak Ca^2+^ oscillations are much more attenuated at high frequency. From these above results, it can be revealed that Ca^2+^ dynamics in response to shear stress and/or ATP stimuli behaves as a low-pass filter. In other words, stimuli at low frequency contribute to significant changes in Ca^2+^ dynamics; on the contrary, pulsatile stimuli at high frequency will be eliminated through the Ca^2+^ signaling system. To verify the wide applicability of the proposed model, instead of sinusoidal pulsatile flows (Figure 6 and Figure 7), an invented CELL-shaped perfusate flow is introduced for the generation of spatio-temporal ATP and shear stress, activated by which the Ca^2+^ response are presented (Figure 8). As shown in Figure 8a, through the transmission within the microchannel, the high frequency components of the CELL-shaped ATP signal, indicated by sharp change in waveform, are greatly attenuated, especially on the side of the perfusion solution (x=3/5W and x=4/5W). For the intracellular Ca^2+^ dynamics (Figure 8b), it is difficult to identify the CELL-shaped form due to the filter effect of both the microchannel and the Ca^2+^ signaling system.

## 4. Discussion

In this study, we developed a theoretical model of intracellular Ca^2+^ responses induced by spatio-temporal ATP and shear stress stimuli within a microfluidic generator. Taking the advantages of microfluidic system for generating biochemical and biomechanical stimuli and its usefulness in probing cellular functions, the proposed model not only demonstrates the capability of the microfluidic generator for creating spatio-temporal and amplitude varying ATP and shear stress stimuli (see Figure 3 and Figure 4), it also reveals how the spatial variation in the combination of ATP and shear stress stimuli affect Ca^2+^ at the single cell level (see Figure 6, Figure 7 and Figure 8). In particular, the spatial-temporal and amplitude variation of stimuli resulted from signal transmission has been characterized, which is commonly encountered in the physiological blood vessels [16,17]. Moreover, we considered both the biochemical (e.g., ATP) and biomechanical (e.g., shear stress) stimuli in both the microchannel and the Ca^2+^ dynamic model, which can mimic approximately the extracellular microenvironment like in the physiological state.

Considering the spatial distribution of temporal ATP and shear stress stimuli, we have analyzed the evolution of ATP concentration distribution within the microchannel (Figure 3) and showed the temporal ATP stimulus (Figure 4a) from the view of culture cells (observing points). The influences of pulsatile frequency and amplitude of introduced pulsatile flow on emerging ATP signals are systematically analyzed (Figure 4b). We found ATP signals of high frequencies are difficult to be generated and transmitted within the microchannel, which are greatly attenuated during transmission. This finding is similar to the low-pass filtering characteristics of microchnnels during the transmission of dynamic biochemical signals [32,33]. Moreover, it is easy to observe that all the presented results are achieved and demonstrated under the conditions of sinusoidal pulsatile flow. Herein, there are two main considerations. One is that sinusoidal signals are the most simple ones from the point of signal processing. Sinusoidal signals are single-frequency in the frequency-domain, which facilitates to analyze their corresponding transmission characteristics. Therefore, sinusoidal pulsatile perfusion solutions are adopted for studying the generation and transmission of sinusoidal ATP stimuli, but sinusoidal signals are not the only suitable waveforms. To verify the wide applicability of the proposed model, a CELL-shaped perfusate flow is introduced into the generation of spatio-temporal ATP and shear stress, activated by which the Ca^2+^ response are presented (see Figure 8). The other reason for using sinusoidal signals is because of the convenience to identify whether nonlinear modulation emerges during signal transmissions (equivalent to mass transport). In this study, the generation and transmission of spatio-temporal ATP signals are regulated by the pulsatile perfusate flow. For this reason, nonlinear modulation effects of pulsatile flows are introduced and lead to the nonlinear distortion of transmitted signals [33]. As indicated in Figure 4a, the emerging ATP signals are not standard sine, which results from the effect of pulsatile flows on the molecule convections and diffusions (see Equation (5)). Therefore, nonlinear modulation effects should be considered when investigating transmissions of biochemical signals within pulsatile flows. Multiple factors should be balanced to reduce and eliminate nonlinear distortion of transmitted signals [33].

Exposed to the spatial combination of ATP and shear stress stimuli, two main response modes of intracellular Ca^2+^ dynamics have been unexpectedly observed (Figure 6 and Figure 7). At the locations of high ATP average, the unimodal Ca^2+^ responses occur, the coupling pulsatile shear stress contributes to the weak oscillations in the basal level. Simultaneously, at the locations of low ATP average combined with pulsatile shear stress, the Ca^2+^ oscillations emerge, wherein the addition of pulsatile ATP stimuli enhances amplitudes and benchmarks of the Ca^2+^ oscillations. Interestingly, this finding is essentially identical with the two regimes of Ca^2+^ dynamics induced by ligand carbachol stimulation [31]: an acute regime triggered at higher ligand concentrations while an oscillatory regime elicited at lower ligand concentrations. This finding proves that spatially varying stimuli will result in the diversity in Ca^2+^ responses. Furthermore, it is easy to infer that the control of spatio-temporal features of stimuli (e.g., average, pulsatile amplitude, and frequency) can modulate the distinct modes of Ca^2+^. As reported in previous studies, unimodal Ca^2+^ responses and Ca^2+^ oscillatory can accordingly regulate and promote cell migration, cell proliferation, and tube formation, critical phases involved in angiogenesis and vasculogenesis [15,19,20]. Thus, the control of Ca^2+^ responses by spatio-temporal physiochemical modulating stimuli would be essential for regulate angiogenesis, which also provides potential alternative strategies to inhibit tumor vascularization [18,20,21].

## 5. Conclusions

In conclusion, based on a developed microfluidic generator of biochemical and biomechanical signals, theoretical modeling has been carried out to investigate the combination effects of spatio-temporal ATP and shear stress stimuli on the intracellular Ca^2+^ dynamics. The proposed mathematical model integrates a module of extracellular signals transmission within the microchannel and a module of intracellular Ca^2+^ dynamics, which mimics the universal conditions for probing Ca^2+^ dynamics based on microfluidic platforms. The results show that the features of pulsatile flows and the transmission characteristics of microchannels greatly affect the generation and transmission of spatio-temporal ATP and shear stress stimuli, resulting in different spatial combinations of stimuli within the microchannel. In response to the various stimuli combinations, the intracellular Ca^2+^ dynamics of cultured cells indicates two Ca^2+^ response regimes depending on the characteristics of stimuli. Overall, the findings of this research provide insights for the intracellular Ca^2+^ dynamics regulated by the combination of spatio-temporal ATP and shear stress stimuli, which have significant implications for understanding the underlying signaling transduction mechanisms and directing cellular behaviors. 

## Figures and Tables

**Table 1:** Dimensions of the microfluidic chip and default parameters used in the sub-model of extracellular ATP transport.

Parameters	Values
*L*	1 × 10^−2^ m
*H*	1 × 10^−3^ m
*W*	5 × 10^−5^ m
*D*	5 × 10^−10^ m^2^/s
$Q_{1}$	1.5 × 10^−10^ m^3^/s
η	1 × 10^−3^ Pa·s
$\phi_{0}$	10 μM
